# RNA Proximity Labeling: A New Detection Tool for RNA–Protein Interactions

**DOI:** 10.3390/molecules26082270

**Published:** 2021-04-14

**Authors:** Ronja Weissinger, Lisa Heinold, Saira Akram, Ralf-Peter Jansen, Orit Hermesh

**Affiliations:** Interfaculty Institute for Biochemistry (IFIB), Tübingen University, 72076 Tübingen, Germany; ronja.weissinger@uni-tuebingen.de (R.W.); lisa.heinold@uni-tuebingen.de (L.H.); saira.akram@uni-tuebingen.de (S.A.); ralf.jansen@uni-tuebingen.de (R.-P.J.)

**Keywords:** RNA–protein complex, proximity labeling, biotin ligase, ascorbate peroxidase, RNA-binding protein, subcellular transcriptomics

## Abstract

Multiple cellular functions are controlled by the interaction of RNAs and proteins. Together with the RNAs they control, RNA interacting proteins form RNA protein complexes, which are considered to serve as the true regulatory units for post-transcriptional gene expression. To understand how RNAs are modified, transported, and regulated therefore requires specific knowledge of their interaction partners. To this end, multiple techniques have been developed to characterize the interaction between RNAs and proteins. In this review, we briefly summarize the common methods to study RNA–protein interaction including crosslinking and immunoprecipitation (CLIP), and aptamer- or antisense oligonucleotide-based RNA affinity purification. Following this, we focus on in vivo proximity labeling to study RNA–protein interactions. In proximity labeling, a labeling enzyme like ascorbate peroxidase or biotin ligase is targeted to specific RNAs, RNA-binding proteins, or even cellular compartments and uses biotin to label the proteins and RNAs in its vicinity. The tagged molecules are then enriched and analyzed by mass spectrometry or RNA-Seq. We highlight the latest studies that exemplify the strength of this approach for the characterization of RNA protein complexes and distribution of RNAs in vivo.

## 1. Introduction

The spatio-temporal control of gene expression is essential to many fundamental biological processes from development and differentiation to synaptic plasticity and memory. This is achieved by coordinating maturation, distribution, stability, and decay of RNAs [1,2]. All these processes involve the formation of different messenger ribonucleoprotein complexes (mRNPs) composed of mRNA and RNA-binding proteins (RBPs). These complexes can be considered as the functional units for posttranscriptional regulation since they do not only contain the information for an encoded polypeptide but also determine the precise spatio-temporal regulation of its translation and thereby facilitate the correct subcellular localization of the translation product [3]. Considering their importance in almost every biological process, it is not surprising that the dysregulation of mRNP complexes contributes to a variety of diseases including cancer and neurodegeneration [4,5]. RBPs as a major component in these complexes can be rather promiscuous. They are not necessarily binding only one specific mRNA, but can interact with various mRNAs as part of different mRNP complexes [6,7,8]. In addition, RBPs can have multiple roles in the regulation of mRNAs such as SR proteins that are involved in splicing, nuclear export, and translation of mRNAs [9]. Genetic and biochemical assays have revealed the function of multiple RBPs, but these assays are often technically limited and do not take into consideration that RBPs need to act in concert to achieve their regulatory role. Thus, characterization of the whole set of proteins that stably or transiently interact with mRNAs should be taken as prerequisite for the elucidation of posttranscriptional regulation. Technological developments made over the last decades like next generation sequencing (NGS), and modern mass spectrometry facilitate such an analysis. Thereby, transcriptome and proteome-wide approaches, coupled to affinity purification of RNP complexes have uncovered several unusual RNA-binding proteins that had previously not been considered as RBPs [10]. The emergence of novel proximity labeling approaches that allow detection of interactions between RNAs and proteins within RNPs in vivo will further advance our knowledge on the constitution of such complexes.

The aim of this review is, after a brief summary of RNA and RBP affinity purification methods, to introduce the rapidly evolving field of in vivo proximity labeling techniques. The review will give an in-depth overview of the application of these techniques in identifying proteins in individual mRNP complexes and characterizing local transcriptomes in cells.

## 2. RIP and CLIP

The classic approach to study RNA–protein interaction is RNA-co-immunoprecipitation (RIP) (protocol summarized in [11]; updated version by Gagliardi and Matarazzo, 2016 [12]). It is based on the simple idea of affinity-purifying an RBP of interest from cell lysate or cell-free preparation to identify its bound RNAs. RIP depends and relies on a specific antibody for the RBP of interest. An alternative if no high-quality antibody is available is the use of tagged proteins like in RNA Bind-n-Seq (RBNS) [13,14].

Although RIP generally relies on the stability of the RNA–protein complex during the purification procedure, improvements like crosslinking of RNA and RBP using UV light (in vivo crosslinking and immunoprecipitation (CLIP)) or formaldehyde have facilitated the characterization of less stably associated binding partners [15] (for an overview, see [16]). Furthermore, UV-crosslinking results in the identification of direct interactions. Crosslinking prior to co-immunoprecipitation also allows more stringent capturing and washing conditions and consequently removing unspecific, not-crosslinked components.

The (standard) CLIP protocol has been modified and optimized over the last decades (reviewed in [16,17]) to overcome problems like UV toxicity, reproducibility, and cross-linking efficiency (e.g., by photoactivatable ribonucleoside-enhanced (PAR)-CLIP [8]) or to allow the mapping of RBP-RNA contact sites with nucleotide resolution (iCLIP [18]). In the commonly used PAR-CLIP variation, the combination of photoactivatable ribonucleosides like 4-thiouridine (4-SU) and less harmful UV-A (365 nm) increases crosslinking efficiency and the rate of thymidine to cytidine transition in the cDNA reads, thereby revealing the RBP binding site. However, PAR-CLIP can only be applied in specific cell lines such as HEK293T as not all cells can efficiently incorporate 4-SU. In order to overcome this restriction, Hinze et al. generated HEK293T based hybrid neuronal cell lines created by cell fusion (Fusion-CLIP), which allowed the use of PAR-CLIP to investigate RBP-binding transcriptomes of neuronal cells [19].

## 3. RNA Affinity Purification

While RIP and CLIP focus on the identification of RNAs associated with specific proteins, other affinity-based methods have been developed to characterize the proteins associated with a specific RNA. The common theme of RNA affinity purification is the capturing or immobilization of an RNA of interest (expressed either in vitro or in vivo) followed by identification of its bound proteins via immunoblotting or mass spectrometry. Two general strategies are used for capturing an RNA of interest: antisense oligonucleotides (ASOs) and RNA aptamer tags.

ASOs can be considered as an equivalent to antibodies for isolating RBPs. They can be designed to hybridize to either a single RNA-specific sequence, a sequence shared by many RNAs like the poly(A) tract in mRNAs or to several regions tiling the RNA of interest. For capturing RNA–protein complexes, biotinylated ASOs are used for the retrieval of the RNA of interest through hybridization, followed by capturing with streptavidin-beads, elution, and identification of the binding proteins. To preserve RNA–protein interactions, crosslinking by UV, formaldehyde or both is usually performed before cell lysis (RNA interactome capture (RIC), [20,21,22,23,24,25]). In order to stabilize ASOs or their binding to the target RNA, LNA (locked nucleic acid) or 2′-*O*-methylated RNA chimeric probes can be used, which also allows to reduce the input material required for capturing [20,26]. The number and the length of ASOs play a crucial role for the efficiency of purification. Single oligo-deoxythymidine (oligo-dT) probes were e.g., successfully used to detect all proteins binding to poly(A) RNA [22,27]. The probing of a specific RNA is achieved mostly using several ASOs (20–120 nucleotides in length) and referred to as ‘tiling approach’. Tiling was successfully applied to identify interacting proteins of lncRNAs including the Xist RNA [24]. However, the use of many probes can result in increased background. A strategy to increase the likelihood to detect specific RBP–RNA interaction involves multiple rounds of purification, which has been applied to the characterization of an oligo-dT bound proteome (serIC, [28]), but also to the interactomes of specific mRNAs like human p27/CDKN1B and yeast PFK2 mRNAs [29]. Another major hurdle to ASO-based capturing is the probe design. It should avoid sequences hidden by interacting proteins or buried in a structured region. This can be done, e.g., by mapping accessible regions in the RNA of interest with RNase H [30].

An alternative way to capture RNAs is to use aptamers encoded in their sequence. Such aptamers are recognized by specific RNA-binding proteins or domains that—when fused to a protein tag, for example—allow pull-down of the RNA for identification and analysis of its interactors. Several different aptamer sequences are currently in use. The MS2 and R17 stem loops, and the BoxB hairpin originate from different bacteriophages and are recognized by the MS2 coat protein (MCP), R17 coat protein, or the phage λ N-peptide, respectively (reviewed in [31]). Furthermore, artificial RNA aptamers have been created that recognize streptavidin [32,33,34] or antibiotics like tobramycin [35] and streptomycin [36]. Before capturing, the in vitro transcribed, aptamer-tagged RNAs are incubated with crude cell lysates to form an RNP with proteins in the lysate [36]. Alternatively, the tagged RNA is immobilized before incubation with the cell extract [35]. Another possibility is the expression of the RNA-aptamer hybrid and formation of RNPs in vivo, followed by cell lysis to isolate the RNPs [37,38]. Two-step aptamer purification, e.g., by combining the tobramycin tag with a PP7 hairpin has improved this method by the reduction of contaminants [39]. However, a potential risk of such time-consuming two-step purification has always been the danger for degradation of the tagged RNA and hence the loss of interactors.

A general consideration is the number of aptamers to be introduced in the RNA, which can range between 1 and 24 [33,40]. Studies using the MS2 aptamer for affinity purification have shown that increasing the number of aptamers up to ten correlated with more efficient pulldown with no further significant improvement using 24 aptamers [41]. However, there have been concerns about the stability or function of the RNA when tagged with MS2, especially when arrays with multiple copies of the aptamer are used (see below; [42,43]).

In general, the higher the affinity between RNA tag and capturing protein or matrix, the harsher the washing condition can be, and the less contaminants should be present in the final product. To date, MS2 aptamer-MCP pair is the most commonly used system for affinity tagging of RNAs since it shows a very good balance of specificity and affinity between RNA and protein [44]. A major advantage is the option to co-express the tagged RNA and the binding protein in vivo to allow formation of RNPs under physiological conditions before cell lysis and pull-down. Fusion proteins of MCP with glutathione-*S* transferase (GST; [40]) or with streptavidin-binding protein (SBP; [45]) have been used for capturing specific MS2-tagged mRNAs. Recently, large scale screens for RNA-interacting proteins have been developed on the basis of this method [41], which demonstrates the potential of the approach.

Despite their utility and strength in analyzing RNA-RBP interaction, RIP, CLIP, and RNA affinity purification methods require cell lysis before capturing and isolating the RNA/RBP of interest. Cell lysis under mild conditions, which is crucial for the preservation of weak interactions and necessary in case no crosslinking can or wants to be performed, can prohibit the analysis of cellular compartments that are not easily solubilized like the nucleus [46]. Furthermore, during cell lysis or subsequent washing steps, interactions can be lost. Crosslinking can be applied to stabilize weak interactions. However, since there is a bias for certain nucleotide-amino acids crosslinking pairs as well as a generally low crosslinking efficiency for double-stranded RBPs, UV-crosslinking will not capture all interactions [47]. When using the more efficient formaldehyde crosslinking, coincidental interactions between RNAs and RBPs might be stabilized due to over-crosslinking [48]. Additionally, independent of prior crosslinking, re-association of RNAs and RBPs after lysis can lead to the detection of false positive interactors [49]. These problems required a solution that avoids the mentioned disadvantages and allows capturing of interactions in vivo.

## 4. Biotin-Based Proximity Labeling Approaches

**Figure 1:**
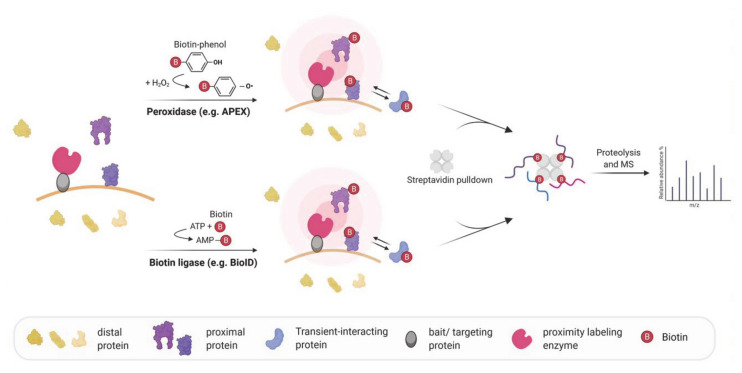
Schematic workflow of peroxidase and biotin ligase based in vivo proximity labeling for mapping molecular interactions. Proximity labeling enzymes fused to a targeting protein are incubated with biotin or a biotin conjugated compound and convert it to a reactive biotin intermediate. Peroxidases oxidize biotin–phenol to reactive phenoxyl radicals using hydrogen peroxide while biotin ligases utilize ATP and biotin to catalyze the formation of reactive biotin-5′-AMP. The biotin intermediate is released from the enzyme and covalently biotinylates proteins in the proximity but not distal proteins. After lysis, the biotinylated proteins are enriched by a streptavidin-pulldown followed by proteolysis and are analyzed by mass spectrometry.

In 2012, proximity labeling was introduced as an alternative approach to the aforementioned techniques to map the molecular interactome in living cells [50]. Predominantly, proximity labeling relies on an enzyme (e.g., biotin ligase or peroxidase) that promiscuously biotinylates and, thereby, covalently labels proteins in its proximity for subsequent isolation and analysis (Figure 1). These enzymes are capable of converting biotin or biotin conjugated compounds into short-lived reactive species which are membrane impermeant [51] and react either with electron-rich amino acids like tyrosine (in case of e.g., phenoxyl-biotin; [52]) or lysine on neighboring proteins (e.g., AMP-biotin; [50]). During the past years, a growing number of different labeling-enzymes, such as ascorbate peroxidases (e.g., APEX, APEX2) and biotin ligases (e.g., BirA*, BioID2, BASU, TurboID, and miniTurbo) have been engineered and used to genetically tag a protein of interest (reviewed in [53]). The experimentally determined labeling radii for proximity-labeling enzymes are estimated within a range of 1–10 nm in living cells [52,54]. In contrast to traditional co-purification strategies, biotin-proximity labeling approaches can identify even weak and/or transient protein–protein interactions without crosslinking in order to stabilize these interactions. Since the proximal interaction partners of a POI are covalently tagged with biotin, harsher extraction and washing conditions can be applied [55].

The first described promiscuous biotin ligase (BirA*) is a mutated version of the *E. coli* biotin ligase BirA [50], a DNA-binding biotin protein ligase, which biotinylates acetyl-CoA carboxylase and acts as a transcriptional repressor for the biotin biosynthetic operon. In the first step of biotin ligation, the enzyme generates biotinoyl-5′-AMP (‘activated biotin’) from biotin and ATP. The reaction intermediate is retained in the active site until it reacts in a second step with a specific lysine residue of its protein substrate (for references see [50,56,57]). The Burke lab [50] took advantage of the reduced affinity of the mutated BirA* enzyme (BirA R118G) for biotinyl-5′-AMP which allows it to dissociate from the ligase and react with proximal proteins, thereby capturing proximal proteins. Since its development, BioID has been widely used to study membrane-bounded compartments like the mitochondrial matrix [56] or large scale structures such as the nuclear lamina [50,58,59] centrosomes [57], cell junctions [58,60], or the composition of protein complexes from infectious pathogens such as *Trypanosoma brucei* [59] and *Toxoplasma gondii* [61].

The main drawback of BirA* is the long labeling time required to achieve sufficient biotinylation (6–24 h), which prevents its use for capturing interactome snapshots [50,62]. Since then, faster enzymes have been engineered, like BioID2 [63], BASU [64], TurboID, and miniTurboID [65]. Both TurboID and miniTurboID enzymes enable biotinylation within 10 min and therefore allow mapping of biological interactions with much higher temporal resolution. However, the higher activity of TurboID can result in the consumption of endogenous biotin, which could potentially result in biotin starvation in cells [65]. Both engineered biotin ligases were successfully applied to different organism including yeast [66], worms [65], flies [65], and plants [67,68].

Peroxidases have been evolved as an alternative to biotin ligases in proximity labeling. Typical representatives of this group of enzymes oxidize biotin–phenol in a hydrogen peroxide dependent reaction to reactive phenoxyl radicals that can react with proximal proteins in short time (usually within one minute; [51,69]). In 2013, the Ting lab has shown that an engineered ascorbate peroxidase APEX, originally used as a tag for electron microscopy [52], can be applied to proximity labeling of the mitochondrial matrix proteome in vivo [51]. The main drawback of APEX however is its low sensitivity when expressed at low levels [70]. This has been overcome by generating APEX2, a variant with much higher activity [70]. APEX2, which allows even shorter labeling times of <1 min facilitates capturing and mapping of interactomes at high temporal resolution and, thus studies of dynamic intracellular interactions. APEX2-based proximity labeling has been successfully applied to study protein signaling networks [71,72]. Furthermore, it has been used to map protein interactions within stress granules [73], the ciliary membrane-associated protein complex [74] or lipid droplets [75], which are compartments that defied standard co-purification techniques. Although all peroxidase-based proximity approaches allow to biotinylate proteins within a short time and high resolution, a major limitation of these enzymes is the potential oxidative stress/toxicity from H_2_O_2_ (reviewed in [53]), which make them challenging to apply in tissues and living organisms.

A complementary proximity labeling approach that has been developed takes advantage of the PafA proximity ligase from bacteria. PafA mediates ligation of a small protein PupE to lysine residues on the surrounding proteins [76]. A caveat of the method is the longer incubation time (several hours), which does not allow to study temporal and spatial snapshots of protein interactions.

A more recent development of proximity labeling enzymes are the split versions like Split-BioID, Split-APEX2, and Split-TurboID [77,78,79,80]. These enzymes consist of two inactive fragments of a proximity labeling enzyme. When fused to two interacting proteins of interest (POI) or proteins in close proximity, the fragments can assemble to an active enzyme, leading to biotinylation only in the vicinity of the interacting POIs. This reduces the chance of biotinylating random proteins before an interaction of the POI with its partner(s) has manifested. In addition, the split versions allow probing for partners in specific protein complexes if the POI is present in several different assemblies [78].

## 5. Proximity Labeling for Mapping Subcellular Transcriptomes

**Figure 2:**
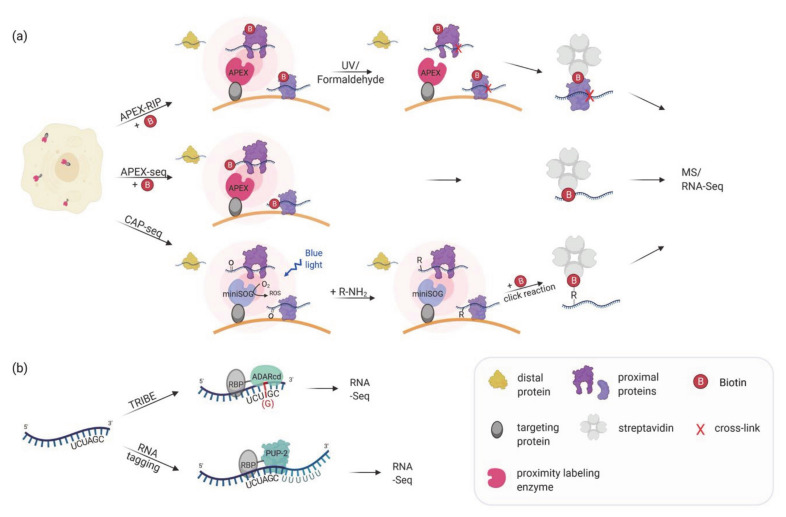
(**a**) Proximity labeling strategies for mapping subcellular transcriptomes. APEX or miniSOG are targeted to a specific subcellular location. In APEX-RIP, APEX catalyzes the biotinylation of proximal proteins, and proximal RNAs are crosslinked by either UV light or formaldehyde treatment. In APEX-seq, APEX directly biotinylates RNA. In CAP-seq, miniSOG oxidizes proximal RNA molecules upon blue light illumination. The oxidized RNAs are crosslinked to an alkylamine probe which can be linked to biotin-azide in a click reaction. APEX-RIP, APEX-seq, and CAP-seq make use of streptavidin-purification of the biotinylated proteins/RNAs followed by MS and/or RNA-Seq. (**b**) In vivo proximity labeling strategies for identifying RNA partners of an RBP. RNA targets of RBPs can be identified by changing the RNA sequence upon the interaction. In TRIBE the RBP of interest is fused to the catalytic domain of ADAR which mediates adenosine to inosine editing (in red) of the interacting RNA(s). The inosine is read as a guanosine when analyzed by RNA-Seq allowing the identification of editing events as A-to-G transition. In RNA tagging the RBP of interest is fused to a poly(U) polymerase (PUP-2) that attaches a poly-uracil chain at the 3′end of interacting RNAs. The uracil tail is then used to identify targets during RNA sequencing.

Following the great success of proximity biotinylation in mapping protein localization and protein–protein interaction, APEX2 was applied to transcriptome mapping (Table 1). In the first attempts, RBPs that were biotinylated by a mitochondrial- or nuclear-targeted APEX2 were used to co-purify their bound RNAs, that had been crosslinked to the proteins by formaldehyde (APEX-RIP; [81]) or by UV (Proximity-CLIP; [82]). To increase the efficiency of UV crosslinking, HEK293T cells were additionally exposed to 4SU in the medium [82]. After enrichment of the biotinylated proteins, the crosslinked RNAs were released and sequenced, whereas the proteins were identified by mass spectrometry (Figure 2a). Proximity-CLIP transcended this analysis by determining not only the type of RNA but in addition the regions protected by the biotinylated RBP [82]. These experiments provided valuable datasets of RNAs in membrane-surrounded organelles but failed to differentiate between RNAs localized at membrane-cytoplasmic interfaces like the ER membrane [81]. The discovery that APEX2 can directly label RNA with biotin, most prominently at guanosine residues [83,84], allowed a direct query of RNAs associated with organelles or located at specific sites (Figure 2a; APEX-seq; [85]). APEX-seq has been applied to detect mRNAs at various cellular locations, among them three subnuclear regions (nucleolus, nuclear lamina, and nuclear pore) [85] or the outer mitochondrial membrane and the mitochondrial matrix [83,85]. In addition, dynamic complexes like the translation initiation complex and repressive RNA granules were investigated [84]. These studies established the first comprehensive cellular atlas of RNA distribution and will facilitate the testing of important biological hypotheses in RNA localization like the translation dependent and independent localization of mRNAs to mitochondria [83,85], or the differential localization of RNA isoforms [86]. In contrast to the direct biotinylation of RNA seen with APEX2, CAP-seq (chromophore-assisted proximity labeling and sequencing) uses miniSOG (for small singlet oxygen generator), a photosensitizer that mediates proximity dependent photo-oxidation of guanine bases in the RNA upon blue light excitation [87] (Figure 2a). Oxidized guanosines are crosslinked to propargyl amine probes taken up by the cells. Following extraction, the RNA is fragmented and biotin is introduced via click reaction (between biotin-azide and the alkyne-conjugated RNA) which allows RNA purification using streptavidin beads. In comparison to APEX-seq, CAP-seq requires longer labeling times (20 min compared to 1 min in APEX-seq), and two steps to conjugate the biotin but offers an alternative labeling approach. Cap-seq was used to identify RNAs at the ER membrane, the mitochondrial matrix and outer membrane [87]. Of the RNAs enriched at the ER membrane, 96% encode for proteins involved in the secretory pathway exemplifying the specificity and the small action radius of the miniSOG.

## 6. Proximity Labeling of RNA–Protein Interactions: Finding the RNA Partners

Proximity labeling methods have also been applied to the characterization of RNAs bound by individual RBPs. This includes methods that are not based on the biotin-streptavidin system but on modifying the sequence of the RNA at or close to the site of protein–RNA interaction (Table 1). In TRIBE (targets of RNA-binding proteins identified by editing) [88], an RBP is fused to the catalytic domain of the RNA-editing enzyme ADAR (ADARcd) that irreversibly deaminates adenosine to inosine on target RNAs bound by the RBP (Figure 2b). During cDNA conversion, the inosine is recognized as guanosine, allowing the identification of editing events as A-to-G mutations when compared to the corresponding DNA sequence. RNAs showing these conversions are considered as potential targets of the tested RBP. However, the edited sites are not completely unbiased due to the preference of ADARcd for adenosines surrounded by 5′ uridines and 3′ guanosines (i.e., a UAG sequence) [98] or surrounded by a double-stranded region [99]. By using an ADARcd with a ‘hyperactive’ mutation that results in increased editing efficiency, the sequence bias was slightly reduced [89]. The method was applied to identify the transcriptome bound by three RBPs (Hrp48, dFMR1, and NonA) in *Drosophila* S2 cells and neurons. The comparison of the RNA targets between neuronal subtypes allowed the identification of cell-type specific RBP-RNA interactions [88,89]. Another labeling technique that results in sequence change of proximal RNAs is poly-uridine tagging [90,91] (Figure 2b). In this case, the POI is fused to the *C. elegans* poly(U) polymerase (PUP-2) that adds a uridine tail to the 3′-end of proximal RNAs. After lysis, RNAs are reverse-transcribed using a primer designed to enrich uridylated RNAs. The resulting cDNA libraries are analyzed using paired-end sequencing for the identity, the number of reads and the number of Us added to the RNA. By fusing PUP-2 to Puf3p in *S. cerevisiae* the authors identified mostly target mRNAs with known Puf3 binding sites [90]. Interestingly, the length of the uridine tail was in correlation with the binding strength of Puf3 to its targets, suggesting that the number of uridines can be used to predict meaningful interactions. The addition of an RNA recognition motif (RRM) and a corresponding targeting sequence to the PUP-2 enzyme was used to characterize the local transcriptomes of ER or mitochondrial membranes in *S. cerevisiae* [92]. However, this method does not provide information on the RNA regions bound by the POI and one can assume that proteins that tether the PUP-2 protein to the 5′ untranslated region (UTR) of their mRNA targets will not allow an efficient oligo-uridylation of their bound mRNAs at their 3′-end. Furthermore, PUP-2 seems to affect cell physiology as its expression resulted in reduced growth rate in yeast [90,91].

## 7. Proximity Labeling of RNA–Protein Interactions: Finding the Protein Partners

**Figure 3:**
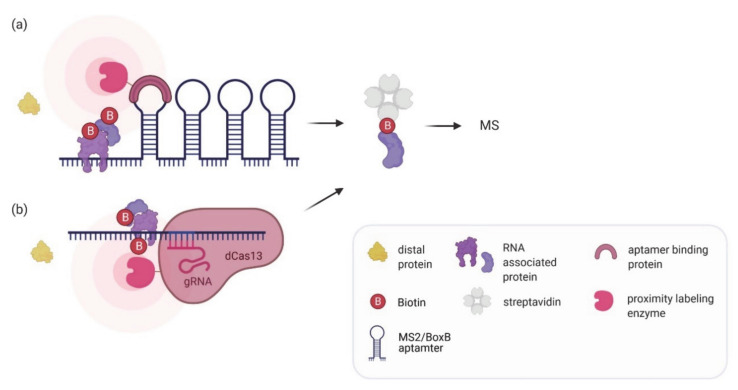
In vivo proximity labeling strategies for identifying protein partners of an RNA of interest. (**a**) The RNA of interest is tagged endogenously or exogenously with aptamers (e.g., BoxB aptamer or MS2 tags) to recruit an aptamer binding protein fused to a labeling enzyme which can biotinylate associated proteins. (**b**) A catalytically inactive dCas13 enzyme fused to a labeling enzyme is targeted to the RNA of interest using a gRNA to biotinylate associated proteins. In both methods biotinylated proteins are enriched by streptavidin pulldown and analyzed by mass spectrometry.

There is an ongoing demand in the field not only to define the set of RNAs bound by a given RBP but also understand what proteins associate with a specific RNA (‘RNA interactome’). In vivo proximity labeling can serve as a good entry point to this problem. By recruiting the labeling enzyme to a specific RNA of interest, its associated proteins will be biotinylated. Two different strategies have been introduced for tethering the labeling enzyme to an RNA: aptamer tagging and CRISPR/gRNA guidance. Aptamer tags like MS2 or BoxB RNA loops have long been used for mRNA localization studies and to follow the dynamics of transcription and translation (reviewed in [100]). Furthermore, they have been applied to affinity purify interacting proteins (see above). RNA proximity labeling using aptamer tagging has been established for overexpressed (RaPID; [64]) or endogenous RNAs (RNA BioID; [62]) (Figure 3a).

In RaPID, a short RNA of interest is flanked by two BoxB aptamers (see below for more details). Its co-expression with a fusion of the BoxB recognizing λ N-peptide and BirA* or BASU allows the biotinylation of proteins bound to or associated with the flanked RNA sequences. RaPID was used to identify proteins that bind to known RNA motifs (e.g., the IRE, TNF-CDE, or PUF RNA motifs [64,101]) and to analyze how mutant RNA motifs affect protein binding. Additionally, by probing the interactome of untranslated regions of the Zika virus genome, an RBP (QKI) that is highly expressed in neuronal progenitor cells was identified as a candidate host protein essential for the Zika virus replication [64]. As suggested by this study, a comparison of the interactome to that of a scrambled RNA and the characterization of biotinylated proteins in cells expressing only the biotin ligase help to reduce the number of false positives. Moreover, the authors suggest performing subsequent analysis like comparison of identified proteins with those reported in the Contamination Repository for Affinity Purification (CRAPome; [102]). Although transient expression of the BASU-λ N-peptide fusion was sufficient for identifying specifically associated proteins for Zika virus, other studies suggest stable genomic integration of the labeling enzyme to increase the signal-to-noise ratio [101]. A potential drawback of the current version of RaPID is that the tagged RNAs are not expressed under their native conditions and therefore are not studied at their physiological concentrations. In contrast, in RNA-BioID [62] the authors characterized the proteome of an endogenous RNA. In this study, the biotin ligase (BirA*) fused to MCP was stably expressed in fibroblasts from a transgenic knock-in mouse line [103] where 24× MS2 aptamers had been inserted into the β-actin gene locus. The modified β-actin RNA, expressed at endogenous levels contains the MS2 aptamers in its 3′ UTR. RNA-BioID not only identified all the RBPs previously reported to bind to β-actin mRNA but also novel functional interactors including FUBP3/MARTA2. However, the major technical hurdle of RNA-BioID is the need to genomically insert the MS2 aptamer array. The development of CRISPR-based knock-in tools might offer a more user-friendly way for this [104]. It is important to note that in RaPID and RNA-BioID, the tested RNAs were highly expressed, either by overexpression from a strong heterologous promoter (RaPID) or due to their high endogenous level of expression (RNA-BioID). This might have facilitated the efficient biotinylation of associated proteins. Thus, it will be interesting to see if these methods can be successfully applied to low abundant mRNAs, especially considering the problem of the observed labeling of non-RNP proteins. In principle, it should be possible to increase the labeling of RNA-associated proteins by increasing the number of aptamers, resulting in the recruitment of more labeling enzymes to the RNA and thus an increase in signal-to-noise ratio. However, as this might affect the function or the stability of the RNA [105,106], a careful examination of the impact of large aptamer arrays on the RNA will be required.

Tethering a biotinylating enzyme to an RNA using the CRISPR system has the advantage of targeting native RNAs at their endogenous expression levels without the need for aptamer fusion (Figure 3b). The tethering occurs with the help of an inactive Cas13 variant (dCas13; [107]). dCas13 can be fused to GFP, enabling imaging of RNA [94], to ADAR2, enabling editing of RNA [108] or to labeling enzymes like APEX2 [95,97] to probe for interacting proteins. A number of Cas13 variants have been used as guiding proteins, e.g., RfxCas13 [93], PspCas13b [97], CasRx [96], and LwaCas13a [95]. Tethering of the fusion protein to RNA can be improved by using a gRNA array instead of single gRNAs [96]. The array is composed of two gRNAs separated by 30 nucleotides to target two adjacent loci on the same transcript. This method (CARPID, CRISPR assisted RNA–protein interaction detection method), was applied to probe the interactome of three lncRNAs (XIST, DANCR, and MALAT1). Interestingly, while the RBPs identified using a single specific set of gRNAs to probe the XIST lncRNA highly correlated between experiments, a lower correlation was found between gRNA sets that probe different regions of the RNA. This implies that CRISPR proximity tools have the potential to study variations in RNA–protein interaction along the target RNA. Multiple gRNAs are also used in a strategy aimed at reducing the number of false positive interactors. Lin et al. targeted a dPspCas13b-APEX2 fusion to the U1 snRNA using three gRNAs [97]. Each of the gRNAs, binding to a different single-stranded RNA region, was expressed in a separate cell line but the interactome data obtained with each gRNA were aligned. This helped to reduce the noise from off-target biotinylation.

The application of tethering proximity labeling enzymes to RNA via dCas13/gRNA requires, however, a thorough optimization. For example, the target sequence of the gRNA has to be single stranded and accessible [109]. Without established knowledge on the folding of the RNA, the effectiveness of gRNAs targeting different regions has to be compared to each other and a non-targeting gRNA. This can be achieved by measuring the ability of each gRNA to knockdown the expression level of the target RNA when co-expressed with the wild-type, active version of the Cas13. However, Han et al. demonstrated that a gRNA targeting the human telomerase RNA (hTR) was not able to target the APEX2 enzyme to the hTR foci in the nucleus although it was efficient in reducing hTR expression [93]. Therefore, the correct delivery of the labeling enzyme to the target RNA should be verified in vivo, e.g., by co-localization experiments. In this study, in order to improve the targeting to hTR foci, a double stranded RNA binding domain (dsRBD) was introduced into the APEX2-dRfxCas13d fusion protein which stabilizes the dCas13/gRNA/mRNA complex [93]. Special care has also to be taken in the design of the fusion construct of dCas13 and the biotinylation enzyme, as it seems important to insert a linker between both parts to uncouple the activities of the two [93]. Additionally, an optimal molar ratio between the fusion construct and the gRNA [97] as well as a stable genomic integration of the fusion protein construct and the control of its expression via an inducible promoter (e.g., via the tet-on system) was reported to be essential for a good signal-to-noise ratio in labeling [93,95].

dCas13-based and MS2-based tethering of a labeling enzyme to the same RNA does not necessarily identify the same set of protein interactors. By comparing the hTR RNA interactome identified by MCP-APEX (i.e., the MCP-APEX fusion recruited to MS2 tagged hTR) with that of dCas13d-dsRBD-APEX2, Han et al. surprisingly found only partially overlapping datasets, with MCP-APEX identifying more potential interactions in total [93]. The difference might, however, simply represent the specific interactome of the subregion targeted by each method. MCP-APEX2 was tethered to the 5′ end of hTR, while dCas13d-dsRBD-APEX2 was targeted to the J2a/3 region 150 nucleotides downstream. An alternative explanation might be based on the limitation of each method. The targeting and RNA-binding by dCas13d, although free from sequence manipulation, is not as stable as the interaction of the MS2 coat protein with its aptamer in the target RNA. As a result of the stable interaction, the MCP resides longer at the RNA which might lead to identifying more interactions, especially transient ones. Moreover, while the insertion of the aptamer might affect the structure of the RNA and thus interaction with certain RBPs, the gRNA might directly compete for crucial protein binding sites on the target RNA.

Similarly to the aptamer methods, the dCas13 based approaches have only been tested for highly abundant or overexpressed RNAs. Therefore, further optimization of RNA-centric proximity labeling is needed to achieve the interactome detection of endogenously, low expressed RNAs.

## 8. Conclusions and Outlook

Proximity labeling has already proven to be a valuable complement to other methods for the analysis of RNA–protein interactions. In the near future, one can expect more and better variations of the approach to address questions in RNP composition and function. The strength of proximity labeling approaches is that they allow the identification of more binding partners, including those interacting transiently, which would not be detected by other methods. However, they do not distinguish between direct binders and proximally located partners. Thus, other techniques like CLIP and RIP still provide valuable information that is complementing results obtained by proximity labeling. This can be exemplified with Xist, a long non-coding RNA that interacts with multiple protein partners. When applied to Xist, proximity-labeling based CARPID identified 73 XIST-interacting proteins, among them 19 that had been previously found as functionally significant binders [96]. However, which of the previously unidentified interactors directly contact the RNA cannot be revealed by this method. Furthermore, at least two previously identified and functionally important Xist RBPs (SPEN and RBM15) were not captured by CARPID [96]. ASO-based affinity purification approaches like RNA antisense purification [24], ChIRP-MS (comprehensive identification of RNA-binding proteins by mass spectrometry) [23], or iDRiP (identification of direct RNA interacting proteins) [110], when combined with UV crosslinking have identified between 10 [24] and 81 [23] proteins, all of which are directly contacting the RNA due to their property of being crosslinked via UV light. Combining proximity labeling with RIP or CLIP on the same protein (MAC-tag; [111]), thus can provide information on direct versus indirect binding and facilitate the identification of true interaction partners. The combination of proximity labeling enzymes with dCas13 will allow to quickly target any RNA of choice for proximity labeling in a variation of cell types. Tethering the fusion protein to the RNA has been improved by including an additional RNA-binding domain, which increases the stability of RNA-dCas13 association [93]. A common problem in proximity labeling applications results from the activity of APEX or biotin ligases even when they have not been tethered to RNA targets which results in increased background. This could be overcome by the use of split versions of proximity labeling enzymes that are independently targeted to the RNA of interest and only assemble to an active enzyme on the RNA. The short biotinylation times of APEX2 (1 min) or TurboID (10 min) will be extremely useful to characterize dynamic changes in the proteome of specific mRNPs in living cells, e.g., before/after nuclear export or before/during translation or localization. New substrates for APEX2 like biotin-anilin or biotin-tyramide [83,84] will improve RNA biotinylation and thus APEX-seq, leading to improved RNA atlases that determine the positioning of mRNAs in the cell [85].

## Figures and Tables

**Table 1:** Proximity labeling methods for the detection of RNA–protein interactions

Proximity Labeling for Mapping Subcellular Transcriptomes						
Method	Description	Model Organism/ Cell Type	Achievements	Strengths	Weaknesses	Ref.
APEX-RIP, Proximity-CLIP	Targeting of a labeling enzyme (e.g., APEX2) to subcellular compartments in order to biotinylate proximal proteins. After crosslinking proteins and RNA, biotinylated proteins are enriched by streptavidin beads and bound RNAs are identified via RNA-Seq.	HEK293T	Identification of compartment specific RNAs, e.g., in the nucleus, cytoplasm, mitochondrial matrix and at the ER membrane.	Proximity-Clip allows identification of RBP-protected regions of RNA targets. Short labeling time. No need of specific antibody.	Limited detection of RNAs in non-membrane bound cellular regions.	[81,82]
APEX-seq, CAP-seq	Targeting of a labeling enzyme (e.g., APEX2, miniSOG) to subcellular compartments or complexes to directly label RNAs. Biotinylated RNAs are enriched by streptavidin beads and identified using RNA-Seq.	HEK293T	Identification of RNAs localized to various locations, including nucleolus, nuclear lamina, nuclear pore, the outer mitochondrial membrane, the mitochondrial matrix, the ER lumen, the ER cytosolic interface and RNA granules.	No crosslinking required. Can identify proximal RNAs in insoluble and open cellular regions. Short labeling time.	Interactomes of individual RBPs cannot be assessed.	[83,84,85]
**Proximity Labeling of RNA–Protein Interactions: Finding the RNA Partners**						
**Method**	**Description**	**Model** **Organism/** **Cell Type**	**Achievements**	**Strengths**	**Weaknesses**	**Ref.**
TRIBE	An RBP of interest is fused to the catalytic domain of the RNA editing enzyme ADAR. ADAR edits target RNAs (A-to-I editing) bound by the RBP, which can be identified by RNA-Seq.	*Drosophila* S2 cells and neurons	Identification of RNAs bound to Drosophila RBPs: Hrp48, dFMR1 and NonA.	No crosslinking required. No specific substrate required for labeling. Can be used to identify the RNA region close to the RBP binding site.	The edited sequence is biased due to the binding and editing preference of ADARcd. ADAR can also edit RNAs in the vicinity but not bound by the RBP. Cannot be used to detect dynamic interactions.	[88,89]
RNA tagging	An RBP of interest is fused to the uridine polymerase PUP-2. PUP-2 attaches an uracil tail to RNAs bound by the RBP which allow their identification by RNA-Seq.	*S. cerevisiae*	Identification of RNA targets of yeast pumilio proteins as well as RNAs localized to ER and mitochondrial surfaces.	No crosslinking required. Counting of added uracil residues allows differentiation of true and false interactors.	Might miss proteins that interact close to the 5′ of the RNA. Can stress cells. Cannot be used to detect dynamic interactions.	[90,91,92]
**Proximity Labeling of RNA–Protein Interactions: Finding the Protein Partners**						
**Method**	**Description**	**Model** **Organism/** **Cell Type**	**Achievements**	**Strengths**	**Weaknesses**	**Ref.**
RaPID, RNA-BioID	An RNA sequence of interest is tagged with either BoxB or MS2 aptamers. The aptamers recruit a viral coat protein fused to a labeling enzyme (BirA*, BASU, APEX2) which biotinylates associated proteins.	HEK293T, huh7, mouse embryonic fibroblasts	Identification of proteins binding various RNA motifs, the UTR of the Zika virus RNA genome, human telomerase RNA, or β-actin mRNA.	Allows identification of weak or transient interactions. High specificity and affinity of MCP or λ N-peptide for their corresponding aptamer. No crosslinking required.	Aptamer insertion might affect RNA function or regulation. Technically challenging to genomically integrate the aptamer cassette at correct location.	[62,64,93,94]
CARPID, dCas13d-dsRBD-APEX2, RPL	Catalytically inactive Cas13 fused to a labeling enzyme (BioID2, BASU, APEX, APEX2) is targeted to an RNA of interest using guide RNAs.	HEK293T	Identification of proteins binding to Xist, MALAT1, DANCR, hTR and U1 snRNA.	No need for changes in target RNA. Probing of different endogenous RNAs can easily be achieved by changing the gRNA. Can be used to probe a specific region on the RNA. No crosslinking required.	Background biotinylation from off-target gRNAs or unbound Cas13-labeling complex possible. Thorough optimization of the Cas13-labeling enzyme construct required.	[93,95,96,97]
